# *Legionella pneumophila* Infection of Drosophila S2 Cells Induces Only Minor Changes in Mitochondrial Dynamics

**DOI:** 10.1371/journal.pone.0062972

**Published:** 2013-04-30

**Authors:** Elizabeth Wen Sun, Monica L. Wagner, Amanda Maize, Doris Kemler, Elisabeth Garland-Kuntz, Li Xu, Zhao-Qing Luo, Peter J. Hollenbeck

**Affiliations:** Department of Biological Sciences, Purdue University, West Lafayette, Indiana, United States of America; University of Louisville, United States of America

## Abstract

During infection of cells by *Legionella pneumophila*, the bacterium secretes a large number of effector proteins into the host cell cytoplasm, allowing it to alter many cellular processes and make the vacuole and the host cell into more hospitable environments for bacterial replication. One major change induced by infection is the recruitment of ER-derived vesicles to the surface of the vacuole, where they fuse with the vacuole membrane and prevent it from becoming an acidified, degradative compartment. However, the recruitment of mitochondria to the region of the vacuole has also been suggested by ultrastructural studies. In order to test this idea in a controlled and quantitative experimental system, and to lay the groundwork for a genome-wide screen for factors involved in mitochondrial recruitment, we examined the behavior of mitochondria during the early stages of *Legionella pneumophila* infection of Drosophila S2 cells. We found that the density of mitochondria near vacuoles formed by infection with wild type *Legionella* was not different from that found in *dotA^–^* mutant-infected cells during the first 4 hours after infection. We then examined 4 parameters of mitochondrial motility in infected cells: velocity of movement, duty cycle of movement, directional persistence and net direction. In the 4 hours following infection, most of these measures were indistinguishable between wild type and *dotA^−^.*infection. However, wild type *Legionella* did induce a modest shift in the velocity distribution toward faster movement compared *dotA^−^* infection, and a small downward shift in the duty cycle distribution. In addition, wild type infection produced mitochondrial movement that was biased in the direction of the bacterial vacuole relative to dotA-, although not enough to cause a significant accumulation within 10 um of the vacuole. We conclude that in this host cell, mitochondria are not strongly recruited to the vacuole, nor is their motility dramatically affected.

## Introduction

The intracellular pathogen *Legionella pneumophila* can infect and replicate in a wide variety of eukaryotic cells, ranging from the freshwater protists that constitute its natural hosts to human alveolar macrophages, where infection becomes manifest as Legionnaires’ disease [9], [10], [11]. When *Legionella pneumophila* infects a cell, it uses its type IV secretion system (T4SS) to introduce into the host cytoplasm a large number of effector proteins [12], [13], [14]. These proteins promote the formation of a niche for bacterial replication by hijacking a wide variety of cell functions, including intracellular signaling and, cytoskeletal structure [15]. Among the specific functions that are altered is the traffic of organelles, particularly elements derived from the endoplasmic reticulum. This results in ER vesicles surrounding the bacterial vacuole and converting it into a compartment that resembles the ER and evades lysosomal fusion [4], [5], [16]. Nested within the first detailed descriptions of the interaction of ER vesicles with the vacuole was the qualitative observation that mitochondria could also be found in the immediate region of the vacuole soon after infection [4], [5], [17], [18], [19]. Since then, others have reported the targeting of L*egionella* effector proteins to mitochondria [20], [21], [22] raising the possibility that infection could alter mitochondrial dynamics [21]. Such an effect on mitochondria could possibly be related to the apparent involvement of the mitochondrial cell death pathway in *Legionella* infection [23], or could be a mechanistically independent, parallel set of events.

However, specific effects of *Legionella* infection on mitochondrial behavior in the host cell have not yet been demonstrated, nor can we yet predict whether any proposed effects are essential or whether they occur in all infectable cell types. Could the redirection of mitochondrial movements underlie either the generation of the *Legionella* replication niche, or a defensive response of the cell? A detailed analysis of this possibility has become more compelling in light of advances in other systems in our understanding of how mitochondrial motility and function are regulated by changes in cellular physiology (e.g., [24]) including in some cases, by bacterial virulence factors [25]. To address these questions, we intended to carry out a screen for host genes involved in *Legionella* effects on mitochondrial motility, using genome-wide RNA interference in *Drosophila* S2 cells [26], [27], [28], [29], a readily-infectable insect cell line [30], [31]. This approach using S2 cells has been successfully applied in a genome-wide screen for host factors in *Listeria* infection [32], in studies of the specific effects of infection on the host cell secretory pathways in *Legionella* [31] and *Salmonella* [33] infection, and in other areas of the life cycle of pathogens [34].

In order first to establish quantitatively the effects of infection on mitochondrial movement, we infected S2 cells with *Legionella* and during the early stages of infection determined several critical parameters, including mitochondrial distribution, velocity, duty cycle and direction of movement. We found that in S2 cells, productive *Legionella* infection does not cluster mitochondria around the vacuole, and has only modest effects on other aspects of mitochondrial behavior. We offer these data as cautionary evidence that not all cell types that can support *Legionella* infection undergo significant changes in mitochondrial dynamics, and as a warning against the attractive idea of screening for the relevant genes in S2 cells.

## Materials and Methods

### Cell Culture and Infection

*Legionella pneumophila* used here were derived from the Philadelphia-1 strain Lp02 [1]. The strains used were: Lp02 pGFP (IPTG-inducible), Lp02 mCherry (we refer to these two as “WT”), Lp03 (*dotA^−^*) pGFP (IPTG-inducible) and Lp03 (*dotA^−^*) mCherry (we refer to these two as “*dotA^−^*” [2]). The LP02 strain encodes one “universal” mitochondrion targeting effector (LegS2) present in all sequenced *L. pneumophila* strains [3]. This strain or derivatives of Philadelphia 1 have been used in previous studies reporting the recruitment of mitochondria by the *L. pneumophila* vacuole [4], [5]. *L. pneumophila* were grown at 37°C on charcoal-yeast extract (CYE) plates or in ACES-buffered yeast extract (AYE) broth [6]. Thymidine (200 µg/ml) was added to support the growth of the GFP thymidine auxotrophical strains derived from Lp02. Chloramphenicol (5 µg/ml) was added to broth to grow the GFP strains. When necessary, IPTG (1 mM) was added to induce GFP expression. To prepare bacteria for infections, all strains were inoculated from fresh patches, grown in AYE broth for about 24 hours to post-exponential phase as determined by increased motility of the bacteria and an optical density of 3.3–3.8.

*Drosophila melanogaster* S2 cells were grown in 1× Schneider’s *Drosophila* Medium with L-glutamine (Life Technologies/Gibco, Grand Island, NY, USA) supplemented with 10% premium, heat-inactivated fetal bovine serum (FBS) from Atlanta Biologicals (Lawrenceville, GA, USA) and 50,000 units/L of penicillin and 50,000 µg/L of streptomycin (Life Technologies/Gibco, Grand Island, NY, USA) at 25°C in 75cm^2^ flasks with 0.2 µm vented caps. The cells were plated 24 hours prior to the experiments at a density of 1.5–2.0×10^5^ cells per 35 mm well on 18 mm round acid-cleaned #1 coverslips coated with 0.5 mg/mL concanavalin A (conA; Sigma, St. Louis, MO, USA) in 6-well plates [7]. For some experiments, cells were grown on 35 mm glass-bottomed dishes with an 18-mm circular hole in the bottom covered by an attached #1 coverglass (MatTek, Ashland, MA, USA), also coated with conA. S2 cells were infected with *Legionella pneumophila* at an MOI of 10 approximately twelve hours after plating. Cells were either infected with *L. pneumophila* having inducible GFP expression (in the presence of IPTG+thymidine at 10 µL/mL of culture medium) or with the strains that have constitutive mCherry expression (see above). For experiments requiring fixation, cells were fixed with 4% paraformaldehyde in PBS at various times after infection, washed in PBS, and mounted for microscopy using Citifluor mounting medium (Ted Pella, Inc., Redding, CA, USA).

### Growth Curves

To determine the time course of *Legionella* growth in S2 cells, cells growing on 18 mm coverslips were infected with WT or dotA*^−^* bacteria constitutively expressing mCherry at an MOI of 5, incubated for 1 hr, and then washed to remove non-internalized bacteria. Cells were then fixed at time points ranging from 1–36 hr post-infection, mounted for epifluorescence microscopy, observed using a Nikon EclipseTi90 microscope equipped with phase contrast and epifluorescence optics and a 60x oil-immersion Plan Apo objective, and 14-bit images were recorded in the red channel using a Photometrics Coolsnap HQ2 camera (Tucson, AZ, USA) or a MicroMAX camera (Princeton Instruments, Trenton, NJ, USA). To determine the number of mitochondrial in vacuoles vs. time, bacterial mCherry fluorescence was quantified from unsaturated 14-bit images using MetaMorph software. A total of 14–67 cells mostly with single vacuoles were examined for each time point.

### Observation and Measurements of Mitochondrial Distribution and Motility

To measure mitochondrial densities in the vicinity of *L. pneumophila* vacuoles, infected cells were stained at various time points after infection with MitoTracker Red CM-H_2_-XROS (MTR; Life Technologies/Molecular Probes, Grand Island, NY, USA) at a concentration of 200 nM for 20 min. Cells were then washed three times with culture medium, fixed and mounted. They were observed as described above, and 12-bit images were recorded in the green (*L. pneumophila*) and red (mitochondria) channels using a Photometrics Coolsnap HQ2 camera (Tucson, AZ, USA). Using Metamorph software (Molecular Devices, Sunnyvale CA, USA), the two channels were overlaid and cells were identified that had small, in-focus vacuoles that were far enough away from other vacuoles and the edge of the cell to obtain a valid measurement. We eliminated large, diffuse, and out-of-focus vacuoles from consideration. The mitochondria channel was sharpened, threshholded and turned into a binary image, and a circle with a diameter of 50 pixels (10.6 µm; area 88.2 µm^2^) was placed around each selected vacuole and the average intensity of mitochondrial fluorescence within the circle was determined. We repeated this analysis using circles with a diameter of 25 pixels (5.3 µm; area 22.1 µm^2^).

Mitochondrial motility was measured from time-lapse sequences taken in live S2 cells. Infected cells in glass-bottomed dishes were washed thoroughly and mitochondria were stained for 30 min with either 200 nM MTR, as described above, or 1 µg/ml of Rhodamine 123 (R-123; Life Technologies/Molecular Probes, Grand Island, NY, USA; [8] followed by three washes with medium and replacement with medium containing 200 nM R-123 immediately before observation. Bacteria within infected cells were identified by observation in either the green channel (for *L. pneumophila* expressing GFP in cells stained with MTR) or the red channel (for *L. pneumophila* expressing mCherry in cells stained with R-123) and phase contrast to insure that bacteria were within vacuoles. We then acquired 200 images in the mitochondrial channel at 2 second intervals; each sequence included a single bacterial fluorescent image and a phase contrast image, both taken immediately before the time-lapse sequence. Image sequences were converted to stacks using Metamorph, exported as AVI files to ImageJ (NIH, Bethesda, MD, USA) and analyzed using the manual tracking plug-in.

### Tracking Mitochondrial Motility

We tracked three to five mitochondria in each cell, gathering the time (sec) and x,y position (µm) for each mitochondrion in each frame. From these data we calculated the distance moved per frame and from that, the average velocity, duty cycle (fraction of time spent moving), and persistence (final displacement from origin divided by total path length). Cell means were calculated for each of these measures and a mean of means was calculated for each experimental condition. To account for very small organelle oscillations as well as small inaccuracies in manual tracking, we disregarded frame-to-frame movements smaller than 0.05 µm/s. To measure the net direction of mitochondrial movements – whether net movement was toward or away from a vacuole – we marked the initial and final positions of 2–15 mitochondria per cell (average = 8.3) in 52 time-lapse sequences. We measured the angle formed by the two lines connecting the bacterial vacuole (“v”) to the mitochondrion’s initial point (“i”), and the initial point to its final point (“f”).

### Statistical Analysis

Statistical tests were performed manually or with GraphPad Prism software (La Jolla, CA, USA). All data distributions were examined by either a 1-sample Kolmogorov-Smirnov test (if n>3) to assess the normality of the data and/or an F-test for equality of variances to determine if parametric statistics could be used. The mean of means from normally distributed data consisting of samples with equal variances were compared using a two-way ANOVA. The mean of means from non-normally distributed data were compared using a non-parametric Friedman two-way analysis of variance. Both analyses tested for significant differences between infection types as well as among time points post infection, with a threshold for significance of α = 0.05. All sets of distributions across the data were analyzed using the non-parametric two-sample Kolmogorov-Smirnov test as the majority of the data did not follow a normal distribution.

## Results

**Figure 1:**
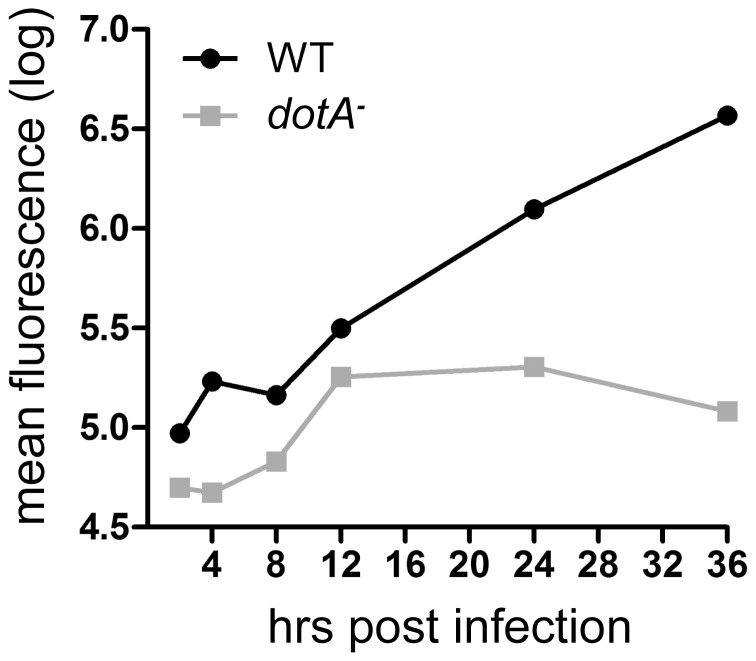
Growth curve for WT and *dotA* *^−^* *L. pneumophila* in *D. melanogaster* S2 cells at 25°C. The replication of mCherry-expressing *L. pneumophila* was tracked via quantitative fluorescence measurements using fixed cells at a range of times after infection, as described in Methods. In this representative experiment, fluorescence of vacuoles in single S2 cells was quantified in an average of 35 cells for each time point, and wild type bacteria and *dotA^−^* mutants show a typical difference in growth curves.

**Figure 2:**
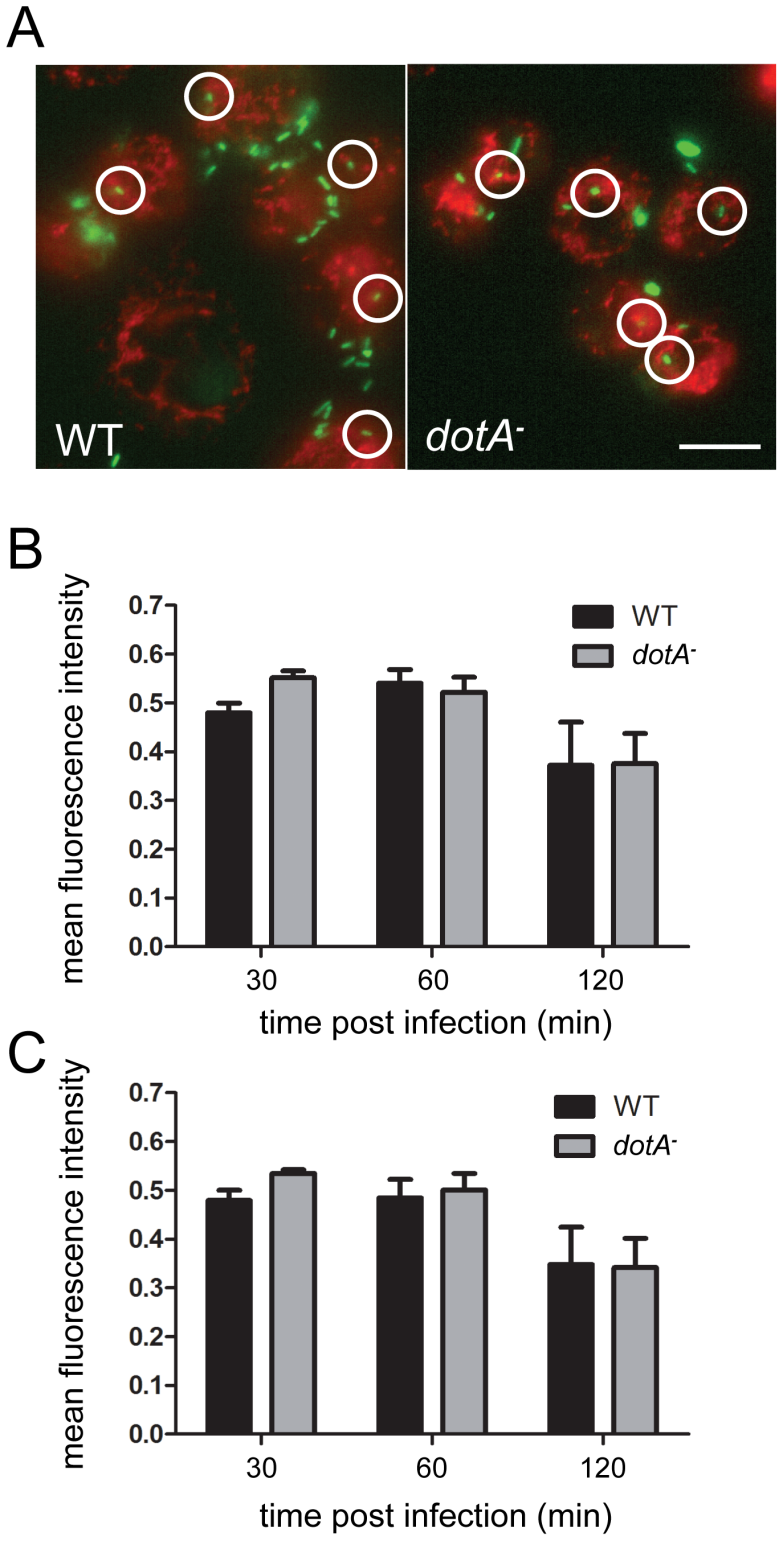
Quantification of mitochondrial distribution near vacuoles formed by WT and *dotA* *^−^* *L. pneumophila*. (A) Representative fluorescent images of S2 cells infected with WT and *dotA^−^* showing placement of 50-pixel ROIs around vacuoles. Mitochondria are stained red with MTR, while *L. pneumophila* are visualized via their expressed GFP. Mitochondrial fluorescence intensities measured within a circular region with a 25-pixel (5.3 µm) diameter (B) or a 50-pixel (10.6 µm) diameter (C) showed no difference in mitochondrial recruitment between the WT and *dotA^−^* infections (N = 3; two-way ANOVA; p>0.05). Error bars = SEM. Scale bar, 20 µm.

Since anecdotal reports have suggested that mitochondria can be found near *Legionella* vacuoles soon after infection, before the log phase [4], [5], we first examined the growth curve for *Legionella* in S2 cells at 25°C. We found that the first four hours after infection under our conditions was a time frame comparable to the pre-log phase time points reported in previous studies (Figure 1). We then infected S2 cells with GFP-expressing wild type (WT) or *dotA^–^* mutant *Legionella* and, after an interval, stained the cells briefly with MitoTracker Red CM-H_2_-XROS, fixed the cultures, and obtained fluorescence images in both red and green channels. Circular regions with diameters of 5.3 and 10.6 µm were drawn around vacuoles and the fraction of each region’s area occupied by mitochondria was determined (Figure 2). Although it was possible to locate specific vacuoles which had a high density of nearby mitochondria, determination of the mean mitochondrial density for many vacuoles at times ranging from 30–120′ post-infection showed no difference between WT and dotA mutants (Figure 2B, C). This indicated that in S2 cells, *Legionella* vacuoles do not concentrate mitochondria in their vicinity.

**Figure 3:**
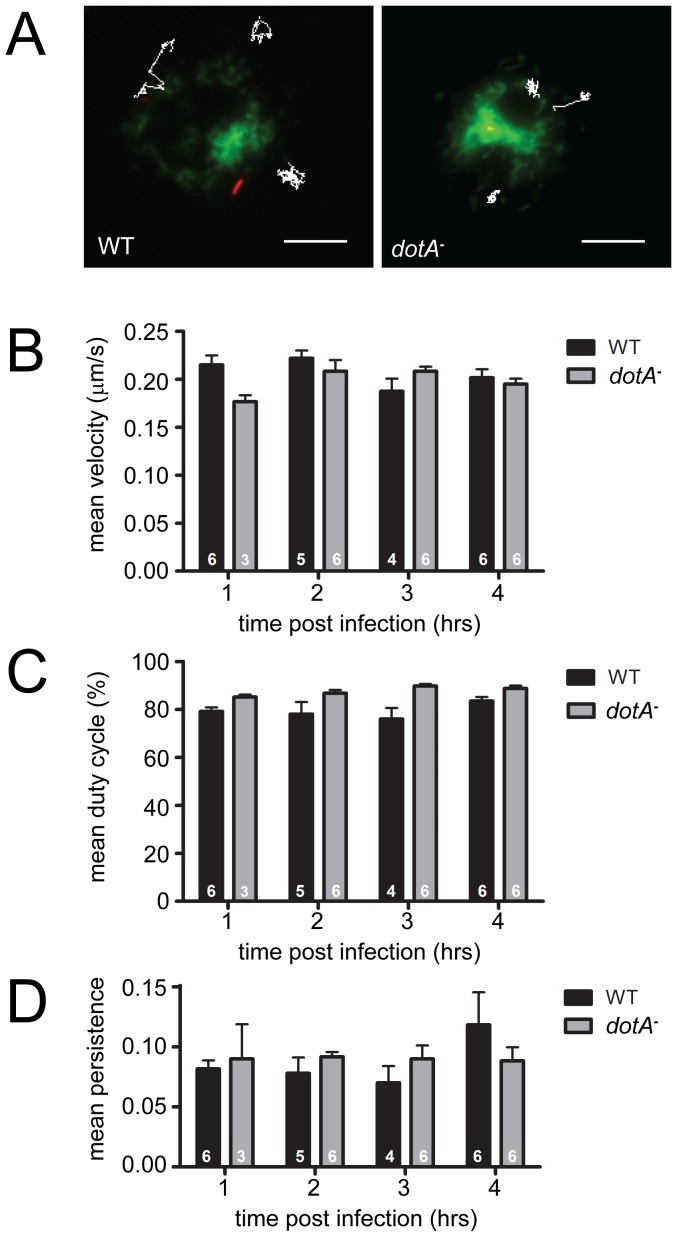
Quantitative measures of mitochondrial behavior in *L. pneumophila*-infected S2 cells. (A) shows images of live S2 cells with mitochondria stained green with R123, and *L. pneumophila* apparent via mCherry expression. The white traces show typical paths for individual mitochondria followed for 140–320 sec in cells infected with WT (left) or *dotA^−^* (right) bacteria. Tracking of individual mitochondrial movements in these cells was used to calculate mitochondrial velocity (B), duty cycle (% of time spent moving, C) and persistence (net displacement/total path length, D) in cells at 1, 2, 3 and 4 hrs post-infection. Analysis of mean values for these measures revealed no significant differences in mitochondrial motility between WT- (black bars) and *dotA^–^*infected (gray bars) cells at any of these time points (Friedman two-way analysis of variance; p>0.05). The white numbers within each histogram bar indicate the number of separate cells in which mitochondria were tracked. Error bars = SEM. Scale bar, 10 µm.

However, mitochondrial motility and distribution could be influenced by infection in a number of functionally-significant ways, so we proceeded to analyze several parameters of mitochondrial behavior in detail over the interval from 1–4 hrs post-infection. Using GFP-expressing WT or *dotA^−^ Legionella* and TMRM-stained mitochondria, we recorded time-lapse movies and tracked mitochondrial movements in infected cells at high spatial and temporal resolution (Figure 3A). From these records we calculated three parameters of movement that have been informative in other systems: mitochondrial velocity during periods of movement; the % of time spent moving, or “duty cycle”; the ratio of net distance moved to total path length, or “persistence.” All three of these were robust measures of mitochondrial behavior with relatively small variance in the means among cells for each treatment. But neither mean mitochondrial velocity, mean duty cycle nor mean persistence varied between WT- and *dotA^–^*infected cells at the 1, 2, 3 or 4 hrs post-infection (Figure 3B–D).

**Figure 4:**
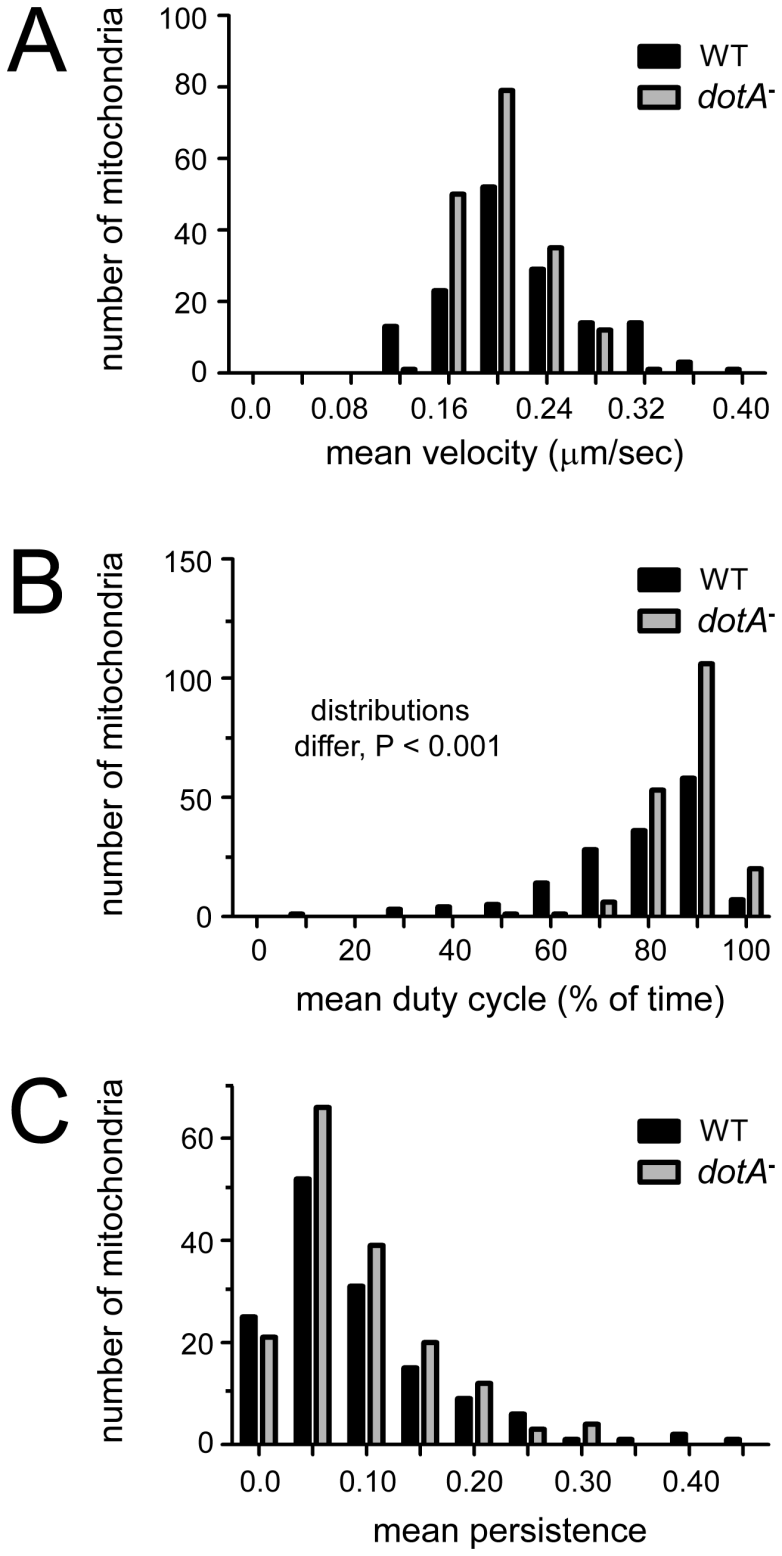
Distributions of mitochondrial motility parameters in WT- and *dotA^–^*infected S2 cells during the first 4 hours post-infection. Mean velocity (A), duty cycle (B), and persistence (C) are shown, data are pooled from 24 (wild type) or 25 (dotA-) movies form 4 separate infection experiments. No differences in distribution were detected that would indicate specific effects of infection on mitochondrial motility. Similar comparisons were made separately for each of the four hourly time points and both bacterial strains (see Figure 5). The majority of the data proved not to follow normal distributions as determined by a one-sample Kolmogorov-Smirnov test. Only the combined active duty cycle distributions (B) were shown to be significantly different, with WT *L. pneumophila-* infected S2 cells showing a reduced mitochondrial duty cycle relative to *dotA^−^ L. pneumophila-* infected cells (P<0.001; Kolmogorov-Smirnov two-sample test).

**Figure 5:**
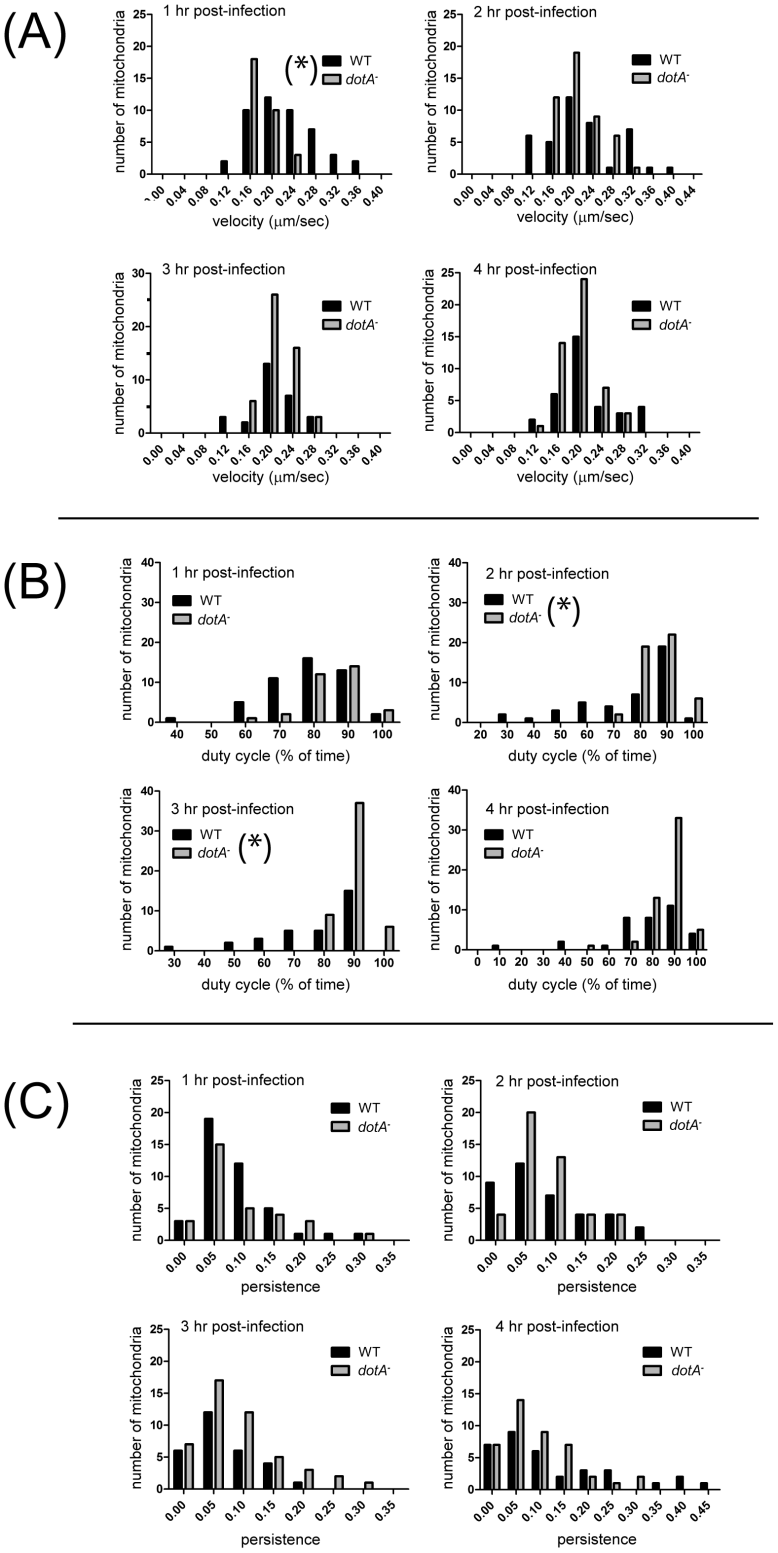
Frequency distributions of mitochondrial motility parameters detailed for each time point. The mitochondrial velocity (A), duty cycle (B), and persistence (C) for WT- and *dotA^–^*infected cells from the experiments summarized in figure 4 are broken out for 1, 2, 3 and 4 hours point post-infection. Most of the data were found not to follow a normal distribution using the 1-sample Kolmogorov-Smirnov test. The only significant differences in mitochondrial motility found between WT- and *dotA^–^*infected cells (indicated by (*)) were (i) the mitochondrial velocity distribution at 1 hr post-infection (P<0.01) and (ii) the duty cycle distribution at both 2 hr and 3 hr post-infection (P<0.025), using the Kolmogorov-Smirnov two-sample test.

Because many moving mitochondria were tracked throughout the cells and the data were pooled, it remained possible that the distribution of any motility parameter might show an outlying tail not detectable in a comparison of means, and perhaps representing the behavior of a subset organelles that were more strongly influenced by the vacuole and the effector proteins emanating from it. Thus, we analyzed the frequency distributions of velocity, duty cycle and persistence for data combined from all 4 time points. The velocity data showed a normal distribution while the duty cycle and persistence data were skewed, one-tailed distributions. For velocity and persistence, we detected no minor outlying peaks indicative of altered behavior by a subset of mitochondria, and the distributions did not differ significantly between WT- and *dotA^–^*infected cells (Figure 4A, C). However, the combined active duty cycle distribution (Figure 4B) showed a tail of lower mitochondrial duty cycles after wild type infection, and the distributions were found to be significantly different, with WT*-*infected cells showing a reduced mitochondrial duty cycle relative to *dotA^–^*infected cells (P<0.001, Kolmogorov-Smirnov two-sample test). To assess the possibility that significant effects on motility occurred within a narrow time window post-infection, we broke out the frequency distributions of all three parameters for all four time points (Figure 5). This analysis showed that the difference we detected in the combined duty cycle data – a reduced % of time moving in infected cells – existed across the early time points, with the differences at 2 and 3 hrs post-infection being most significant (P<0.025; Figure 5B). Thus, WT infection was associated with a modest decrease in mitochondrial duty cycle from 1–4 hrs post-infection. The detailed mitochondrial velocity distributions showed that at 1 hr post-infection a subset of mitochondria moved faster in WT- than in *dotA^–^*infected cells (Figure 5A), with the 1 hr distribution differing, WT vs *dotA^−^*, significantly overall (P<0.01). Thus, WT infection generated a modest increase in the velocity of a subset of organelles, but this did not persist much past 1 hr post-infection. For all other time points and parameters, the detailed distributions of motility behavior were indistinguishable between WT- and *dotA^–^*infected cells.

**Figure 6:**
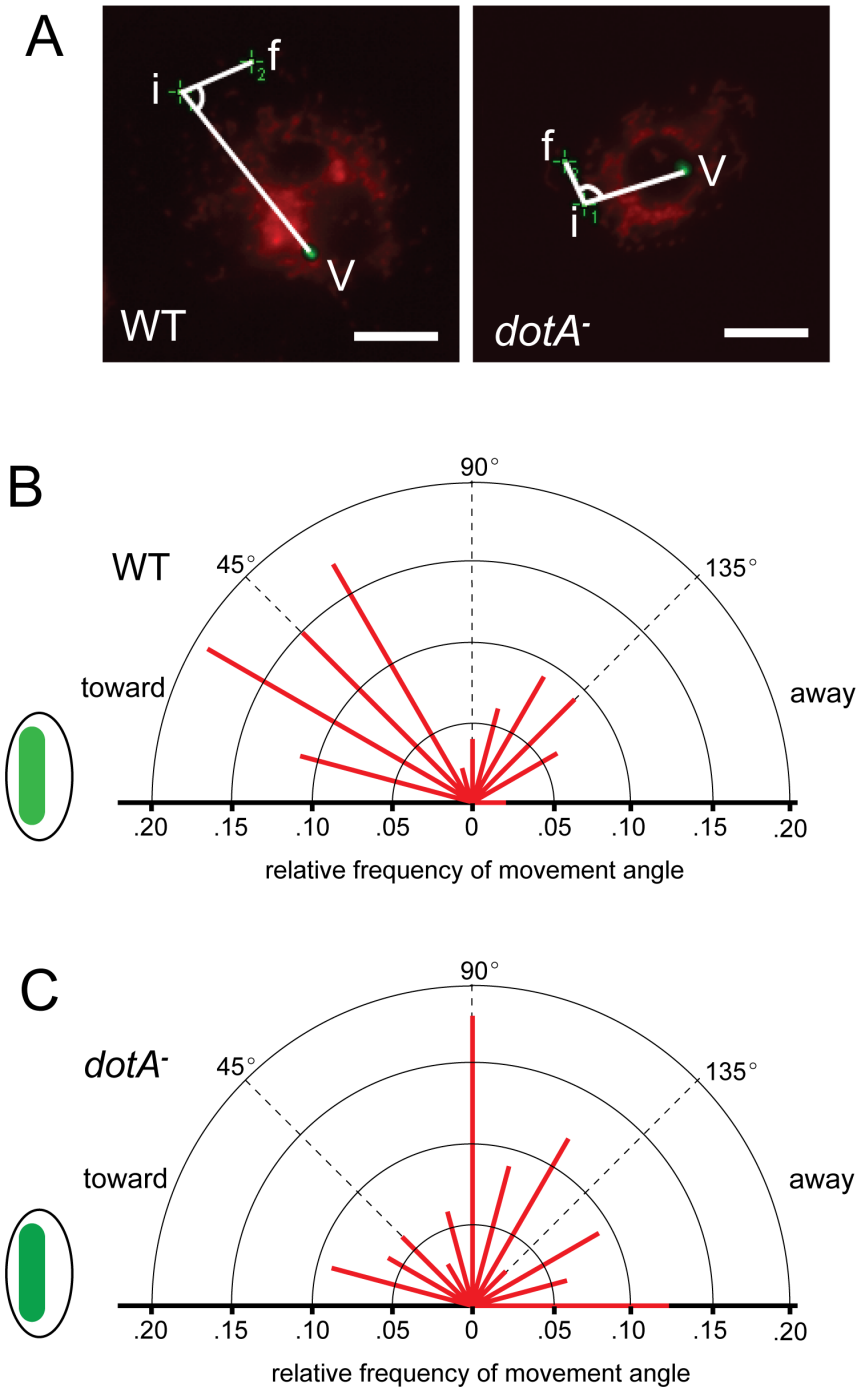
Fluorescent images illustrating the angles tracked for MTR-labeled mitochondrial movement in GFP expressing WT and *dotA* *^−^* *L. pneumophila-* infected cells (A). “V” represents the vacuole containing the bacterium, “i” indicates the initial position of the mitochondrion, and “f” indicates the final position of the mitochondrion. The relative frequency of the angle of mitochondrial movement towards or away from the vacuole at 1 hour post infection of WT and *dotA^−^ L. pneumophila*-infected S2 cells are shown in (B, C). The data are represented as the relative frequencies (in 15° bins) as indicated by the length of the lines displayed over 180°. The origin of all radiating relative frequency lines corresponds to the initial position “i,” as shown in (A), for each mitochondrion. All angle measurements considered included only the smaller θ between the three points; thus allowing all angles to be plotted on a semi-circle. Non-moving mitochondria were excluded from the analysis. The distribution of directions of movement was significantly different between the WT and *dotA^−^* infected cells at 1 hr post-infection (p<0.01; Kolmogorov-Smirnov two-sample test). Scale bar, 20 µm.

Although the effects of the vacuole on the kinetics of mitochondrial motility were limited, it remained possible that the direction of mitochondrial movements was affected – not enough to cluster them significantly in the immediate region of the vacuole, but perhaps enough to shift the distribution of mitochondrial function in the infected cell. To test this, we tracked mitochondrial movements for 2 min and used the first and last point of each track to determine the angle of the net movement relative to the vacuole (Figure 6A). We found that the distribution of net movement angles was different in WT- versus *dotA^–^*infected cells: Net movement toward the vacuole (vacuole-initial point-final point angle <90°) was more frequent in WT-infected cells (64% of mitochondria) than in *dotA^–^*infected cells (30%) (Figure 6B, C).

It was also possible that although mitochondria did not cluster in the region of a vacuole, and although cell-wide effects on motility were small, the vacuole might nonetheless alter the motility of only the subset of mitochondria that were in close proximity to it. To test this, for every mitochondrion that we tracked in cells 1 hr post-infection, we plotted each of the three motility parameters (velocity, duty cycle and persistence) versus distance from the vacuole. We did not observe any significant correlations between motility and distance (r^2^<0.2 for all three parameters), nor did we observe a subset of mitochondria very close to the vacuole whose behavior was discrete from the population as a whole (data not shown). Thus, the modest effects we observed on mitochondrial motility seemed to be found rather uniformly throughout infected S2 cells.

## Discussion

This is the first study to look quantitatively at the response of host cell mitochondria to *Legionella* infection. Based on descriptive studies [4], [5], we expected mitochondria to accumulate in the region of the vacuole in S2 cells. This result would have been interesting and important from the standpoint of mitochondrial function and the cellular environment for bacterial replication. Instead, we found no significant accumulation of mitochondria near the vacuole and only modest effects on mitochondrial motility, despite looking exhaustively at mitochondria in infected S2 cells: examining means and frequency distributions to detect changes in behavior of small subsets of mitochondria, and also examining possible effects of proximity to the vacuole on mitochondrial behavior. The latter was important, since organelles responding to the vacuole and its secreted proteins could be expected to show effects that varied with distance. However, despite looking quantitatively with high spatial and temporal resolution, we saw only minor reductions in duty cycle and increases in velocity for a sub-population of mitochondria at early time points. Although we carried out basic comparisons between WT-infected and uninfected S2 cells, our detailed analyses were focused entirely on comparing WT-infected to *dotA^–^*infected cells. This was because we sought to discern specific organelle behaviors caused by *Legionella* effector proteins rather than non-specific effects simply due to presence of a phagosome.

We were motivated to study this issue because there has been descriptive evidence for mitochondrial co-localization with *Legionella* vacuoles from electron micrographs, mainly in two studies from 30 [4] and 10 years ago [5], and indeed the mitochondrial “response” to infection is assumed to be widespread and has been enshrined in review cartoons. However, no quantitative and controlled experimental approach has been applied to the question. Nonetheless, additional work has indicated indirectly that mitochondria in host cells might respond to infection.

First, there is now evidence that among *Legionella*’s secreted proteins are some that could affect mitochondrial function. Indeed, some substrates of the Dot/Icm transporter are known to specifically target mitochondria. For example, the *Legionella* effector LegS2, probably acquired from a protozoan during phylogenetic development and shared among all sequenced *L. pneumophila* strains [3], is targeted to mitochondria in COS-7 cells, where it may be involved in sphingolipid metabolism [20]. In addition, a mitochondrial carrier protein has been identified in *Legionella* that is introduced into the cytoplasm of macrophages by the T4SS and causes a dominant negative mitochondrial phenotype when expressed in yeast [22]. This protein is dispensable for intracellular bacterial replication [22] and is absent in strain LP02, suggesting it has a nonessential role, if any, in mitochondria recruitment by the bacterial vacuole. Such plasticity of Dot/Icm substrates among different strains of *L. pneumophila* has been well documented [3], [35]. It is likely that a group of as yet unidentified effectors shared among these strains are responsible for modulating the activity of the mitochondrion, including its trafficking in certain host types. The *Legionella* chaperonin HtpB has been shown to alter mitochondrial distribution and actin organization [21], although this result is difficult to integrate into a model of secretion and cytoplasmic signaling since the chaperonin was reported to act on mitochondria not when it was expressed in the cytoplasm but only from outside of the cell. Indirect evidence also comes from studies of *Legionella* infection of *Dictyostelium*, in which expression of proteins of the mitochondrial electron transport chain is up-regulated upon infection [36] but where, conversely, the success of bacterial replication is enhanced by mitochondrial dysfunction [19]. Finally, although less relevant to *Legionella*, the cytoplasmic pathogen *Listeria monocytogenes* causes a transient effect on the mitochondrial fission-fusion balance in infected HeLa cells [37].

This wide and varied range of results begs a question: what possible roles could mitochondria play in the course of infection, and how each could be evaluated? Could mitochondrial recruitment or changes in motility benefit *Legionella*, or the host, or have an indirect relationship to the events of infection that benefits neither? And what other mitochondrial functions are good candidates for modulation by *Legionella’*s secreted proteins? Mitochondrial recruitment to the vacuole could, for example, benefit *Legionella* by bringing increased mitochondrial function into the region of the vacuole – production of ATP, local buffering of Ca^2+^, or synthesis of important lipids. Some effects on function could also be achieved by more complex effects on mitochondrial motility, such as modulating fission-fusion balance. If infection does affect mitochondrial motility and distribution, such effects could also be indirect. For example, association between the ER and mitochondria could bring mitochondria to the region because ER vesicles have been directed there, particularly if functions in which the ER and mitochondria interact, such as Ca homeostasis or lipid synthesis, are modulated by infection. It also remains possible that a mitochondrial response to infection might be part of the host cell’s innate immune response, inhibiting infection through changes in metabolism or even the production of reactive oxygen species (ROS) that compromise the ability of *Legionella* to replicate.

This raises the issue of the range of mitochondrial functions that are candidates for modulation by *Legionella*. In addition to changes in motility, metabolism and ROS production, the mitochondrial role in apoptosis is a potential target for both suppression and stimulation during the course of infection. We are currently evaluating these functions in infected cells. A final, more speculative point is the possible interaction of mitochondria with *Legionella*’s secretion system. Since the T4SS is derived from the conjugation apparatus used in horizontal transfer of genetic material between bacteria, it is not unreasonable to ask whether mitochondria retain any capacity to interact with it, and whether such interactions can stimulate, modulate or damage mitochondria in host cells.

We were drawn to this question because we work on the regulation of mitochondrial distribution, motility and function in neurons [24]. The hijacking of ER vesicle traffic by *Legionella* is clearly important for successful infection, and the possibility that the traffic of other organelles might be modified – particularly those with profound functional implications for the cell, such as mitochondria – is of both pathogenic and cell biological interest. Genome-wide RNAi screens in Drosophila S2 cells have helped to determine the constellation of genes involved in a number of processes (eg, [27]), including questions about microbial pathogenesis [33], [34] and, specifically, aspects of *Legionella* pathogenesis [31]. Thus it seemed very attractive to approach mitochondrial involvement in infection using this method. Our data warn against pursuing it in this system. While several non-physiological host cells also fail to reorganize mitochondria in response to *Legionella* infection, some physiological hosts cells do (our unpublished data). Future studies will reveal more comprehensively how mitochondria are involved in the host cell response to infection.
